# Left upper lobectomy for lung cancer with a subaortic anomalous left brachiocephalic vein: a case report

**DOI:** 10.1186/s44215-026-00266-1

**Published:** 2026-06-19

**Authors:** Ryuhei Sakata, Yukihiro Yoshida, Masaya Yotsukura, Tomohiro Haruki, Shun-ichi Watanabe

**Affiliations:** Department of Thoracic Surgery, National Cancer Center Hospital, Tokyo, Japan

**Keywords:** Anomalous left brachiocephalic vein, Aortopulmonary window, Lung cancer, Mediastinal lymph node dissection, Video-assisted thoracoscopic surgery, Recurrent laryngeal nerve

## Abstract

**Background:**

A subaortic anomalous left brachiocephalic (innominate) vein is a rare systemic venous anomaly. Although usually asymptomatic, when it traverses the aortopulmonary window it may obscure the operative field and increase the risk of vascular injury during left upper mediastinal procedures, including station 4 L lymph node dissection and exposure or control of the left pulmonary artery.

**Case presentation:**

A man in his seventies presented with a 4.2-cm solid mass in the left upper lobe (S3b) and an enlarged station 4L lymph node. Endobronchial ultrasound-guided transbronchial needle aspiration (EBUS-TBNA) of station 4L demonstrated carcinoma consistent with non-small cell lung cancer (NSCLC). Given resectable single-station N2 disease and pre-existing interstitial lung disease with a usual interstitial pneumonia (UIP) pattern, a surgery-first strategy was selected. Preoperative contrast-enhanced computed tomography (CT) with three-dimensional reconstruction demonstrated an anomalous left brachiocephalic vein coursing along the left lateral side of the aortic arch and then beneath the aortic arch, superior to the left pulmonary artery, and crossing the aortopulmonary window before draining into the superior vena cava (Takada pattern b/c). Video-assisted thoracoscopic left upper lobectomy with systematic mediastinal lymph node dissection was performed. Early identification of the left vagus nerve enabled safe proximal tracing to the left recurrent laryngeal nerve. En bloc dissection of the station 4L and station 5 lymph node regions and left pulmonary artery management were completed without neural or vascular injury. The ductus arteriosus ligament (DL) could not be identified intraoperatively, suggesting a retroductal course (Takada pattern c).

**Conclusions:**

Preoperative identification of a subaortic anomalous left brachiocephalic vein on contrast-enhanced computed tomography (CT) with three-dimensional (3D) reconstruction, together with early identification of the vagus and recurrent laryngeal nerves and careful pulmonary artery management after establishing a safe dissection plane, may facilitate safe dissection in the aortopulmonary window and help prevent neural or vascular injury.

## Background

An anomalous left brachiocephalic (innominate) vein is an uncommon congenital systemic venous anomaly in which the vein courses beneath the aortic arch rather than along its usual anterior route. In adult CT-based cohorts, anomalous left brachiocephalic vein has been reported to be extremely rare, with incidences ranging from 0.03% to 0.062% [7]. It is often associated with congenital heart disease or aortic arch anomalies; however, it is also detected incidentally in adults undergoing contrast-enhanced computed tomography (CT) for unrelated indications [1–3, 7].

Takada et al. classified subaortic courses of the anomalous vein according to its relationship to the aortic arch, the ductus arteriosus ligament (DL), and the pulmonary artery [1]. When the anomalous vein traverses the aortopulmonary window, it becomes clinically relevant to thoracic surgeons because it may restrict the operative corridor and increase the risk of vascular injury during left upper mediastinal dissection, including station 4 L lymphadenectomy, as well as during pulmonary artery exposure and control [1, 4–6].

### Case presentation

A man in his seventies was found to have a 4.2-cm solid mass in the left upper lobe (S3b). Computed tomography (CT) and fluorodeoxyglucose positron emission tomography (FDG-PET) showed enlarged lymph nodes at stations 4 L and 11 and bilateral lower-lobe reticulation with honeycombing, consistent with a usual interstitial pneumonia (UIP) pattern. No apparent FDG uptake was observed in the station 5 or 6 lymph node regions. Endobronchial ultrasound-guided transbronchial needle aspiration (EBUS-TBNA) of station 4 L demonstrated carcinoma consistent with non-small cell lung cancer (NSCLC). Given resectable single-station N2 disease and underlying interstitial lung disease with a UIP pattern, a surgery-first strategy with systematic lymphadenectomy was selected after multidisciplinary discussion. A preoperative biopsy of the primary lung lesion was not performed because EBUS-TBNA had already confirmed mediastinal nodal malignancy, and definitive histology and molecular testing were planned using the resection specimen.

**Fig. 1:**
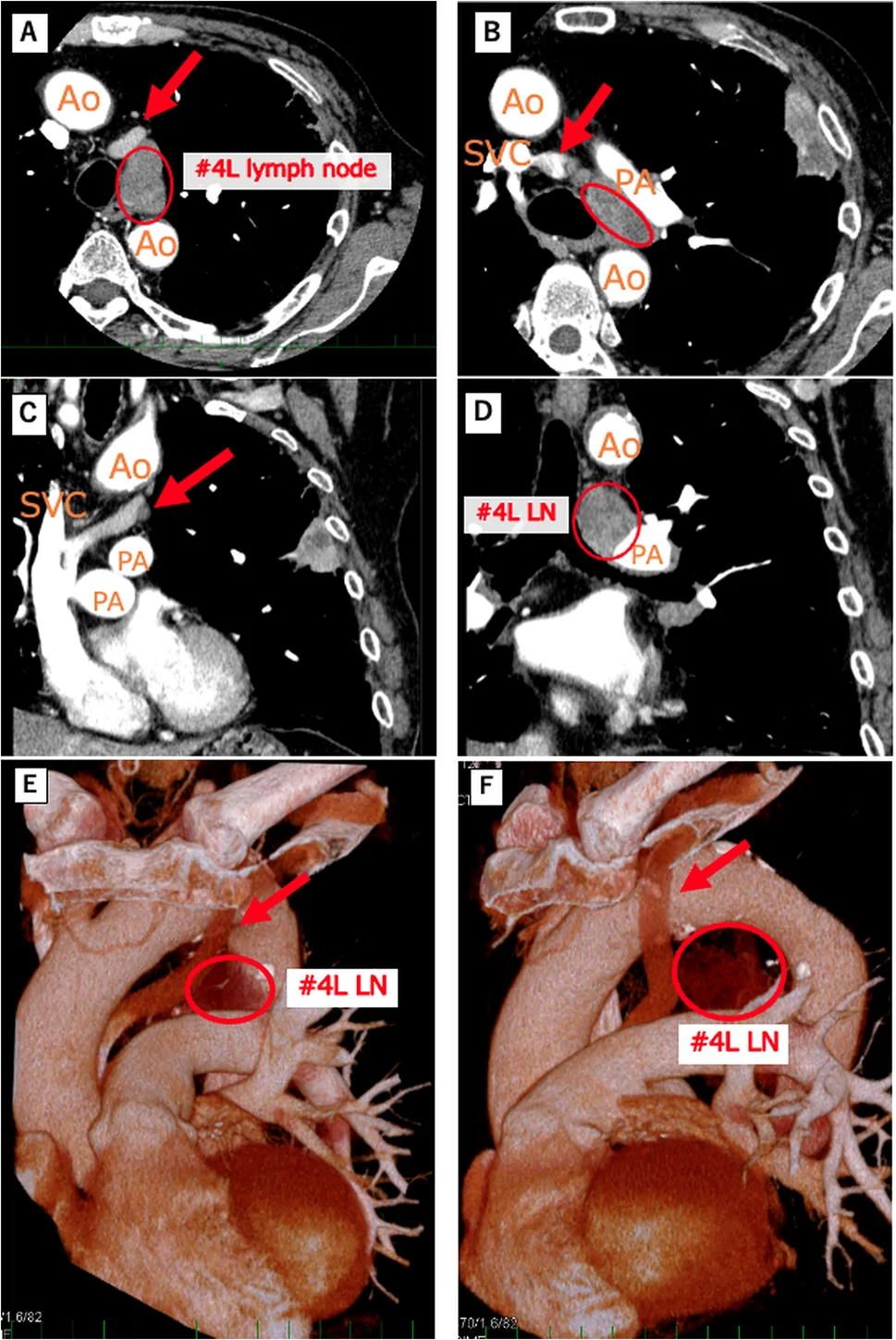
Preoperative contrast-enhanced computed tomography (CT) and three-dimensional (3D) reconstruction. (**A**, **B**) Axial CT images and (**C**, **D**) coronal CT images demonstrate a subaortic anomalous left brachiocephalic vein coursing through the aortopulmonary window, in relation to the enlarged station 4 L lymph node and left pulmonary artery. (**E**, **F**) Enlarged three-dimensional reconstruction images show the anomalous left brachiocephalic vein crossing the aortopulmonary window and the station 4 L lymph node. The anomalous left brachiocephalic vein is indicated by arrows, and the station 4 L lymph node is highlighted by an oval. Ao, aorta; PA, pulmonary artery; SVC, superior vena cava

Preoperative contrast-enhanced computed tomography (CT) with three-dimensional (3D) reconstruction demonstrated an anomalous left brachiocephalic vein coursing along the left lateral side of the aortic arch and then beneath the aortic arch, superior to the pulmonary artery, and crossing the aortopulmonary window before draining into the superior vena cava. Based on these findings, the course was considered consistent with Takada pattern b/c [1, 3, 6] (Fig. 1A–F).

**Fig. 2:**
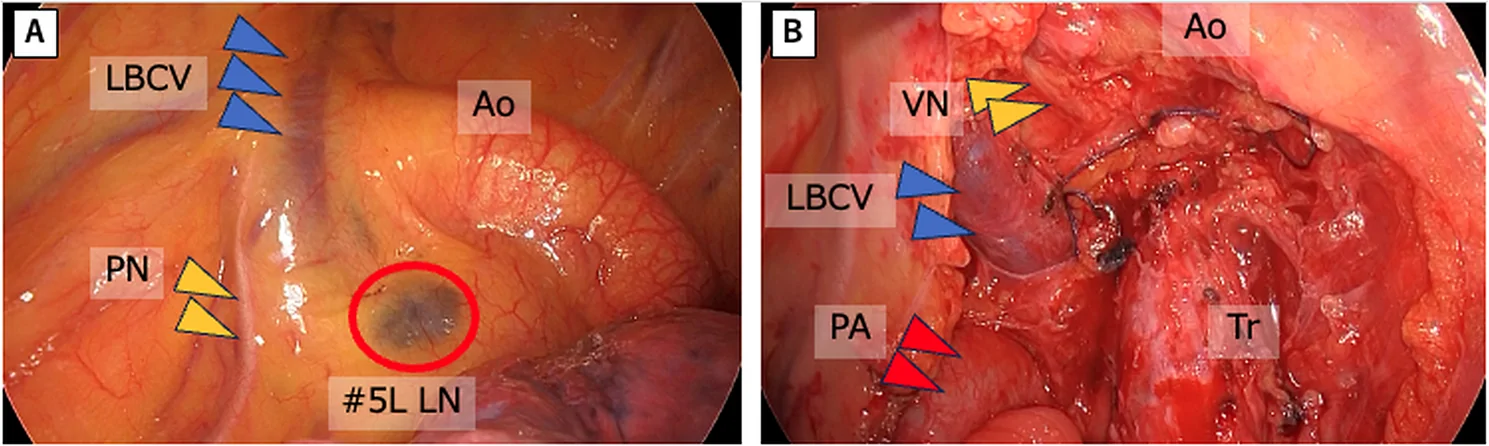
Intraoperative views of the aortopulmonary window. (**A**) Before mediastinal lymph node dissection, an enlarged station 5 lymph node is indicated (circle) beneath the mediastinal pleura. (**B**) After en bloc dissection of the station 4 L and station 5 lymph node regions, the subaortic anomalous left brachiocephalic vein is exposed in the aortopulmonary window. The spatial relationship among the anomalous LBCV, left pulmonary artery, vagus nerve, and recurrent laryngeal nerve was confirmed before deep dissection in the aortopulmonary window. Red arrowheads indicate arteries, blue arrowheads indicate veins, and yellow arrowheads indicate nerves. Ao, aorta; LBCV, left brachiocephalic vein; PA, pulmonary artery; PN, phrenic nerve; Tr, trachea; VN, vagus nerve

Video-assisted thoracoscopic surgery (VATS) left upper lobectomy with systematic mediastinal lymph node dissection was performed. A 10-mm 30-degree thoracoscope was inserted through a camera port placed in the seventh intercostal space on the midaxillary line, and a 7-cm anterolateral utility thoracotomy was created through the fourth intercostal space. The subaortic anomalous vein occupied the aortopulmonary window, restricting the working space during station 4 L lymphadenectomy and exposure of the left main pulmonary artery. The anomalous LBCV coursed along the left lateral side of the aortic arch and then beneath the aortic arch, passing through the aortopulmonary window in close proximity to the station 4 L and station 5 lymph node regions and left pulmonary artery. The mediastinal pleura was opened on the dorsal mediastinal side of the hilum to identify the left vagus nerve, which was then dissected proximally to allow safe identification of the left recurrent laryngeal nerve. Although careful dissection was required because of the restricted operative field in the aortopulmonary window, identification of the recurrent laryngeal nerve itself was not technically difficult after the vagus nerve had been identified. The ductus arteriosus ligament (DL) could not be identified intraoperatively. En bloc dissection of the station 4 L and station 5 lymph node regions was performed after confirming the spatial relationship among the anomalous LBCV, left pulmonary artery, vagus nerve, and recurrent laryngeal nerve. No suspicious lymph node was identified in the station 6 region intraoperatively (Fig. 2).

The operative time was 2 h 29 min, and blood loss was 117 mL. No vascular injury occurred. The patient was discharged on postoperative day 4. Pathological examination revealed large cell carcinoma with mediastinal nodal metastasis, pT2bN2M0 (stage IIIA; TNM 8th edition). Pathologically, metastasis was identified only in one station 4 L lymph node.

## Discussion

A subaortic anomalous left brachiocephalic vein is rare and may be overlooked unless contrast-enhanced computed tomography (CT) is carefully reviewed. In adults without congenital heart disease, CT-based series have reported incidences of 0.03% and 0.062%, underscoring the rarity of this anomaly in adult thoracic practice [7]. Previous radiologic series and review articles have emphasized the importance of recognizing this anomaly for operative planning and for avoiding misinterpretation as mediastinal lymphadenopathy [1–3, 5, 7]. Its relevance to thoracic surgeons becomes greatest when the anomalous vein traverses the aortopulmonary window, because it can narrow the operative corridor and complicate both station 4 L lymphadenectomy and exposure or proximal control of the left pulmonary artery. This issue is particularly important in patients with clinically suspected station 4 L disease, in whom both complete nodal dissection and safe vascular management may be required.

In the present case, preoperative contrast-enhanced CT with three-dimensional (3D) reconstruction clarified the anomalous venous course and allowed us to anticipate the restricted anatomy of the aortopulmonary window. On the basis of this anatomical understanding, we adopted a structured intraoperative strategy. The vagus nerve was identified early and traced to the recurrent laryngeal nerve to establish reliable landmarks before deep dissection in the aortopulmonary window. Pulmonary artery manipulation was then deferred until a safe dissection plane had been established. We believe that this sequence contributed to the safe completion of both station 4 L lymphadenectomy and left upper lobectomy without neural or vascular injury.

**Fig. 3:**
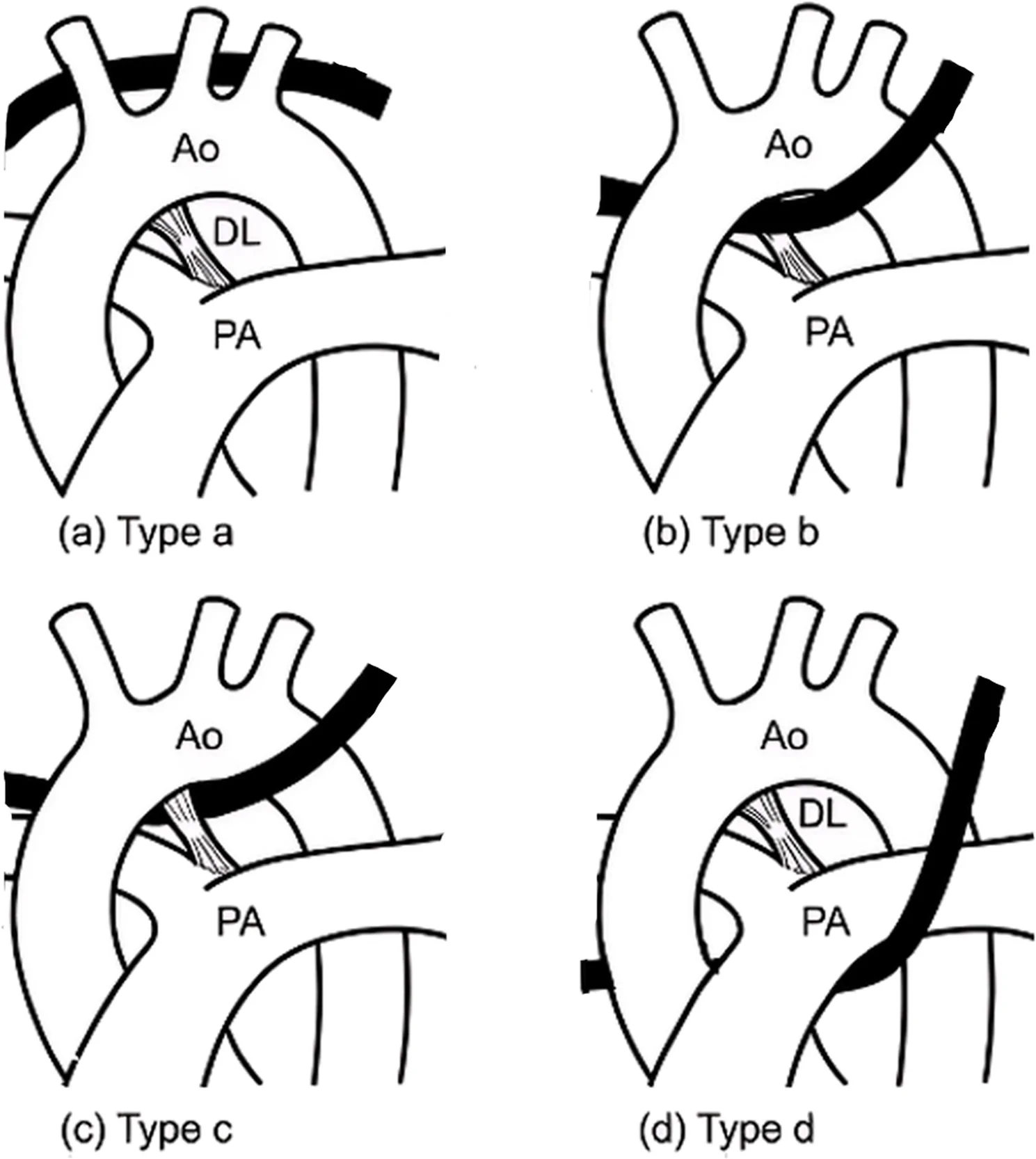
Schematic illustration of the Takada classification of a subaortic anomalous left brachiocephalic vein (LBCV). The thick black line indicates the course of the anomalous LBCV. (**a**) Type a: the anomalous LBCV passes posterior to branches arising from the aorta. (**b**) Type b: the anomalous LBCV passes beneath the aortic arch (Ao) and anterior to the ductus arteriosus ligament (DL). (**c**) Type c: the anomalous LBCV passes beneath the aortic arch (Ao) and posterior to the DL (retroductal course). (**d**) Type d: the anomalous LBCV passes beneath the aortic arch (Ao) and inferior to the pulmonary artery (PA). Ao, aorta; PA, pulmonary artery; DL, ductus arteriosus ligament (ligamentum arteriosum). Schematic drawn by the authors based on the classification described by Takada et al. [1]

Precise subtype classification may be difficult in adults because the ductus arteriosus ligament (DL) is not always identifiable on imaging or intraoperatively. In CT-based series, type b has been reported as the most common type according to Takada’s classification [3, 7]. In our case, failure to identify the DL in the expected location suggested a retroductal course, corresponding to Takada pattern c; however, definitive confirmation was not possible [1, 4] (Fig. 3). Only a limited number of reports have described lung resection in patients with a subaortic anomalous left brachiocephalic vein [8]. Our case adds practical operative detail by showing how preoperative anatomical recognition and stepwise intraoperative dissection can help manage left-sided lung cancer when the anomalous vein constrains the aortopulmonary window.

Treatment selection for clinical stage IIIA non-small cell lung cancer remains complex. In the present patient, interstitial lung disease with a usual interstitial pneumonia pattern increased concern for treatment-related pneumonitis, which supported a surgery-first strategy with careful perioperative management [9]. Although this background influenced treatment selection, the principal message of this case is surgical: when a subaortic anomalous left brachiocephalic vein traverses the aortopulmonary window, safe surgery depends on accurate preoperative anatomical recognition and disciplined intraoperative sequencing.

## Conclusion

In conclusion, a subaortic anomalous left brachiocephalic vein traversing the aortopulmonary window can compromise the operative field during left upper mediastinal procedures, including station 4 L lymphadenectomy and pulmonary artery control. Preoperative evaluation with contrast-enhanced computed tomography, with or without three-dimensional reconstruction, is valuable for identifying this anomaly and planning a safe approach. In such cases, early identification of the vagus and recurrent laryngeal nerves provides reliable landmarks for dissection, whereas pulmonary artery management should be undertaken only after a safe dissection plane has been established. This stepwise strategy may help prevent neural and vascular injury when left upper mediastinal dissection and pulmonary artery management are required within a constrained aortopulmonary window.

## Data Availability

Not applicable. No datasets were generated or analyzed for this case report.

## Abbreviations

CT: Computed tomography
DL: Ductus arteriosus ligament
EBUS-TBNA: Endobronchial ultrasound-guided transbronchial needle aspiration
FDG-PET: Fluorodeoxyglucose positron emission tomography
LBCV: Left brachiocephalic vein
NSCLC: Non-small cell lung cancer
UIP: Usual interstitial pneumonia
VATS: Video-assisted thoracoscopic surgery

## References

[1] Takada Y, Narimatsu A, Kohno A, et al. Anomalous left brachiocephalic vein: CT findings. J Comput Assist Tomogr. 1992;16(6):893–6. 1430437 10.1097/00004728-199211000-00012

[2] Chern MS, Ko JS, Tsai A, Wu MH, Teng MM, Chang CY. Aberrant left brachiocephalic vein: CT imaging findings and embryologic correlation. Eur Radiol. 1999;9(9):1835–9. 10602959 10.1007/s003300050931

[3] Chen SJ, Liu KL, Chen HY, Chiu IS, Lee WJ, Wu MH, et al. Anomalous brachiocephalic vein: CT, embryology, and clinical implications. AJR Am J Roentgenol. 2005;184(4):1235–40. 15788602 10.2214/ajr.184.4.01841235

[4] Gerlis LM, Ho SY. Anomalous subaortic position of the brachiocephalic vein (innominate): a review of published reports and report of three new cases. Br Heart J. 1989;61(6):540–5. 2667595 10.1136/hrt.61.6.540 PMC1216712

[5] Berko NS, Jain VR, Godelman A, Stein EG, Ghosh S, Haramati LB. Variants and anomalies of thoracic vasculature on computed tomographic angiography in adults. J Comput Assist Tomogr. 2009;33(4):523–8. 19638843 10.1097/RCT.0b013e3181888343

[6] Nagashima M, Shikata F, Okamura T, et al. Anomalous subaortic left brachiocephalic vein in surgical cases and literature review. Clin Anat. 2010;23(8):950–5. 20830788 10.1002/ca.21046

[7] Yamamuro H, Ichikawa T, Hashimoto J, et al. Congenital anomalies of the left brachiocephalic vein detected in adults on computed tomography. Jpn J Radiol. 2017;35(10):597–605. 28849388 10.1007/s11604-017-0673-4

[8] Nakamura R, Inage Y, Iwasaki K, Yumoto T, Yuzawa K, Ueki H. Left anomalous brachiocephalic vein in a patient with right lung cancer. Ann Thorac Surg. 2013;96(1):307–9. 23816083 10.1016/j.athoracsur.2012.11.030

[9] Jiang A, Lu H, Zhao R, Yuan J, Chen D. The impact of pre-existing interstitial lung disease on radiation and checkpoint inhibitor pneumonitis in lung cancer patients: a systematic review and meta-analysis. Ther Adv Med Oncol. 2025;17:17588359251338624. 40351325 10.1177/17588359251338624 PMC12064914

